# Identifying and characterising sources of variability in digital outcome measures in Parkinson’s disease

**DOI:** 10.1038/s41746-022-00643-4

**Published:** 2022-07-15

**Authors:** George Roussos, Teresa Ruiz Herrero, Derek L. Hill, Ariel V. Dowling, Martijn L. T. M. Müller, Luc J. W. Evers, Jackson Burton, Adrian Derungs, Katherine Fisher, Krishna Praneeth Kilambi, Nitin Mehrotra, Roopal Bhatnagar, Sakshi Sardar, Diane Stephenson, Jamie L. Adams, E. Ray Dorsey, Josh Cosman

**Affiliations:** 1 Birkbeck College, University of London, London, UK; 2 Bill and Melinda Gates Foundation, Seattle, WA USA; 3 Panoramic Digital Health, Grenoble, France; 4 Takeda, Deerfield, IL USA; 5 Critical Path Institute, Tucson, AZ USA; 6 Radboud University Medical Center and Radboud University, Nijmegen, The Netherlands; 7 Biogen, Cambridge, MA USA; 8 Roche, Basel, Switzerland; 9 Alnylam Pharmaceuticals, Cambridge, MA USA; 10 University of Rochester, Rochester, NY USA; 11 Abbvie, North Chicago, IL USA

**Keywords:** Health care, Biomarkers

## Abstract

Smartphones and wearables are widely recognised as the foundation for novel Digital Health Technologies (DHTs) for the clinical assessment of Parkinson’s disease. Yet, only limited progress has been made towards their regulatory acceptability as effective drug development tools. A key barrier in achieving this goal relates to the influence of a wide range of sources of variability (SoVs) introduced by measurement processes incorporating DHTs, on their ability to detect relevant changes to PD. This paper introduces a conceptual framework to assist clinical research teams investigating a specific Concept of Interest within a particular Context of Use, to identify, characterise, and when possible, mitigate the influence of SoVs. We illustrate how this conceptual framework can be applied in practice through specific examples, including two data-driven case studies.

## Introduction

Intensified by the implications of the COVID-19 era^1^, Digital Health Technologies (DHT) are widely recognised as a promising complementary element in the clinical assessment of Parkinson’s disease (PD). A key enabler is the wider availability of smartphones and wearables which offer the opportunity to enable monitoring of disease progression in daily life^2–4^. More frequent assessments can provide better insight into episodic disease features such as motor fluctuations, freezing of gait, and falls, while avoiding observation bias^5^. Yet, to operationalize DHTs as drug development tools, they must meet the key challenge of regulatory acceptability, so that digital outcome measures can be established as evidence for medical product development.

Yet, despite progress made toward regulatory maturity of DHTs, their use in clinical research is not yet fully accepted^6^. Common challenges in the adoption of DHTs include small study samples, samples that do not reflect accurately the characteristics of the target population, lack of a normative data set, feature selection bias, failure to replicate results due to differences in sensor placement and calibration, and lack of transparency in the use of analytical techniques^7^. When employed at home, DHTs enable higher-frequency data collection compared to traditional clinical assessments. However, this setting can also introduce significantly greater variability between subjects, for example due to differences in apartment size, and within subjects, for example due to differences in room temperature and the presence of family members. Furthermore, studies incorporating machine learning (ML) and artificial intelligence (AI) based approaches in particular, are at high risk of providing overly optimistic results due to feature selection bias when a large number of post hoc candidate features are considered in a relatively limited sample^8^. This is especially relevant when cross validation methods are used to assess performance on a single modestly-sized dataset.

In this context, a key consideration is how to identify, characterise, and when possible, mitigate the influence of key sources of variability (SoVs) introduced by the measurement process and to understand their influence against changes to symptom severity and disease progression. This challenge is further intensified by the heterogeneous nature of PD expression leading to high intra- and inter-study variability.^9^ For example, to address a specific hypothesis, selection of study subjects is often biased (e.g., early disease only) and therefore typical variability associated with disease heterogeneity is reduced within a specific study. Ideally, to address this issue multiple data sources would be needed. However, the availability of data sets using DHTs is limited and analyses on limited data can artificially increase the degree of explained variability, leading to bias in insights and predictions. Variability introduced by the measurement process, such as differences in the placement of a wearable, lack of control of the home environment, device software upgrades in the course of a study, or the accuracy of the specific model of sensor used, must be set into the context of normal variability in the subject and how this is impacted by PD.

This paper provides a conceptual framework to assist clinical research teams to identify, characterise and mitigate the influence of key SoVs introduced by the measurement process and contrast their effect against changes due to PD severity and progression. We illustrate how this conceptual framework can be applied in practice through multiple examples including two case studies developed using pilot data contributed by the co-authors.

## Results

The primary focus in the design of a clinical investigation is the clinical event or measurable characteristic of PD that is to be assessed and the proposed trial population^10^. For example, the clinical research team would typically identify appropriate outcome assessments, preferably a Performance Outcome (PerfO) when DHTs are considered, or digital biomarkers that are meaningful in the specific Context of Use. In this regard, Taylor, et al.^10^ outlined the importance of distinguishing between data- and patient-centric approaches: While either approach could influence the assessment of motor experiences in PD due to a variety of SoVs, appropriate mitigation strategies such as test-retest studies are recommended. Next, alternative DHTs should be assessed in terms of design and operation and their suitability considering the education, language, age and technical aptitude of the population targeted. The goal is to establish that the particular device choice is fit-for-purpose for the specific clinical investigation including its physical characteristics; to validate its outputs including data format and accuracy; and to validate the selected digital outcome measure and the method of its calculation. Last but not least, the clinical research team must provide objective evidence that the selected technology and associated measurement process accurately assesses the clinical event or characteristic in the proposed participant population. To this end, investigation of SoVs should be considered a core ingredient in developing comprehensive and convincing evidence of validation, ideally through the quantitative assessment of their influence against performance changes due to Parkinson’s.

### Identifying and characterising SoVs in the DHT measurement processes

The design of the clinical protocol and of the measurement process may affect SoVs differentially: for example, free-living vs. clinical setting assessments or the choice between active or passive tests. Each alternative may introduce different trade-offs that need to be considered separately. While variability is likely to be greater in a free-living environment, this setting may be preferable to provide greater insight into activities of daily living, therefore better represent the patient’s individual capacity, and be less biased as a comparison against a clinical scale. Indeed, a key distinction among DHTs for the assessment of PD is between: (i) active assessments, in which the subject is prompted to perform a particular set of movements, activities, or tasks at a particular time and for a specific duration; and, (ii) passive assessments, where data is sampled continuously by a recording device worn on the body without prompting or other types of direct interaction with the subject. The most common approach in conducting active assessments today involves the use of a smartphone app such as mPower, HopkinsPD, OPDC, Roche, and cloudUPDRS^3,4,11^. These apps typically guide the subject through a series of tasks that are often associated with specific sub-items of Part III motor assessments of the MDS-UPDRS, a rating scale often used clinically to assess for severity of PD features^12^ (for examples of typical movements during active testing cf. video at http://www.updrs.net/help/). In any of these studies, the app uses one or more of the smartphone sensors such as accelerometer, gyroscope, microphone, magnetometer, and touch screen, to record measurements associated with the specific task. Apps typically also collect contextual information such as the time of medication intake, self-assessments of well-being, or answers to clinical questionnaires such as the PDQ-8, and may incorporate cognitive assessment tasks such as the Stroop test^13^. In contrast, passive monitoring is used to assess patients based on activities of daily living. Consequently, passive monitoring approaches induce less patient burden compared to active monitoring tasks and support continuous monitoring over days. Moreover, continuous passive monitoring can be used to assess response fluctuations of dopaminergic medication as well as the detection of episodic features, e.g., freezing of gait and falls. Finally, passive monitoring approaches typically fix the placement and orientation of the wearable device, and thus multiple devices are required to assess left and right and upper and lower body movements.

#### Precept 1: Establish SoVs in active vs passive measurement processes

Measurement processes for active and passive measurements introduce different SoVs. Active tests require the subject to actively engage with a device following a specific schedule. Measurements are influenced by clinical protocol-dependent variations in the number, frequency, and the exact timing of the active tasks performed by the study subjects. Due to the prescribed nature of these measurements, missing data may also occur, which is less likely in a passive measurement process.

In contrast, due to the lack of environmental context in passive testing, it is often challenging to accurately identify the specific task or activity undertaken by the subject during data recording. For example, a type of movement such as riding a bicycle, may not always be adequately recognized. Because it is not always possible to establish ground truth through observation, the practical alternative is often to infer context using machine learning techniques^14^. A common approach is to employ pre-trained models to classify sensor data into activities such as sitting, walking, cooking and so forth. However, such computational methodologies are still in relatively early stages of development, especially at population level, and can accurately account only for a small proportion of all daily activity. Furthermore, manual annotation of activities is still required for validation of algorithm performance. AI and ML algorithms trained to detect the types of activity of clinical interest may then perform poorly when passively collected data contains many types of activities that were not in the original training and validation data. The largest to date published longitudinal study of daily activity achieved less than 30% accuracy across subjects with the best individual accuracy of less than 65%^15^. The key characteristics of active and passive approaches are summarized in Table 1.

**Table 1:** Comparison between active vs. passive digital assessments.

Active assessments	Passive assessments
Proactive interaction with associated patient burden	Relatively unobtrusive operation with low patient burden
Specific duration of observation	Continuous measurement
Relatively small volume of data	Relatively large volume of data
Known context of data collection constrained to specific movements	Unknown context of data collection affected by unknown external factors
Can be combined with clinical assessments	Predominately unsupervised operation in a non-clinical setting
Effort-intensive to conduct longitudinally	Longitudinal observation by default
Episodic assessment of specific tasks	Real-life functioning of subjects
SoVs can be more easily recognized and examined systematically. Controlling of SoVs is feasible (see also precept 2).	SoVs are more difficult to identify and typically are more difficult to replicate. Controlling of SoVs is less feasible.

#### Precept 2: Identify SoVs associated with acquisition, management, and analysis within the measurement process

The measurement process for both active and passive approaches can be separated into three distinct stages and key SoVs relevant to each stage can be identified (cf. Table 2). Detailed descriptions of each factor included in the three distinct phases, namely data acquisition, management, and analysis, are included in a companion paper derived by the work of the Critical Path for Parkinson’s Consortium 3DT Working Group^16^ on metadata standards and reported in ref. ^17^.

**Table 2:** Mapping SoVs across different measurement process phases.

Data acquisition	Device/sensor configuration
	Assessment tasks and duration
	Sensor positioning and orientation
	Environment
	Schedule of assessment
	Precision and frequency
	Meta-data: device specification, data acquisition setup, file naming, hardware, and software versioning
Data management	Source data file transmission
	Data receipt notification
	Data quality control (missing data, malfunctioning device or sensor, erroneous sampling, erroneous transmission, corrupted storage, timing errors)
	Adverse events assessment
	Notification of data quality concerns and troubleshooting
Data analysis	Signal processing method used for feature extraction
	Signal processing architecture: edge, cloud, or hierarchical/hybrid
	Documentation of algorithms and implementation

The detail of these phases is device and application-specific, for example in some applications, significant data analysis is done on the wearable device itself.

#### Precept 3: Characterise low-, medium- and high-impact SoVs

The third element of the conceptual framework characterises SoVs as low, medium, and high impact relative to the risk they present in terms of their potential to cause harm on the ability of digital outcomes to measure clinically relevant aspects of PD if they are not dealt with appropriately. Low-impact SoVs are those that are well-understood and mitigation strategies are readily available, often already incorporated in devices or as a standard feature of data processing software. Medium impact SoVs are well understood and effective means for their control and mitigation are widely available and in common use, for example, through the application of appropriate algorithms or user experience design approaches. Compared to low-impact SoVs, they require more attention, and their mitigation should be specifically addressed in study design but appropriate mitigation measures but do not require extensive further investigation. Finally, high impact SoVs are those that present a significant risk to influence the performance of the digital outcome measure of interest, their characteristics are not adequately documented and quantified, thus mitigation approaches are not readily available or require further validation. Note that the concept of impact in this context incorporates the risk to reduce the fidelity of the measure as well as the maturity and robustness of mitigation methods. However, it excludes the degree of complexity of the mitigation technique applied; for example, low-impact SoVs may still require the implementation of advanced computational methods. Moreover, as discussed later in this paper, note that the precise risk of harm by a particular SoV is only possible to fully quantify within a specific Context of Use.

### Low-impact SoVs

Following the above categorisation, thermal effects introducing variability in accelerometer measurements would be classified as a low-impact SoV due to the fact that their effect is well-understood^18^ and, indeed, the vast majority of good quality commercial devices incorporate a temperature sensor which is used to adjust the data output accordingly. Gravity is also considered a low-impact SoV for acceleration measurements when the sensor orientation can change. In this case, the effect of gravitation on magnitude estimation can be removed by the application of a standard high-pass filter on the 3-axis signal. When movement directionality along each axis is required, an algorithm such as an L_1_-trend filter can be used^19^.

A further example of low-impact SoV is the audio capture quality of current smartphones: Grillo et al.^20^ tested a variety of devices and found negligible variability in the calculation of common acoustic voice measures using a commercial software tool including many of those widely used in PD^21^. However, they discovered considerable overall differences when alternative algorithms were used to calculate the same measure, suggesting that software artifacts present a higher impact SoV. In this case, algorithm implementation would be considered a medium-impact SoV as it was still possible to mitigate software variability by adjusting for the observed trend across calculated measures, which followed similar patterns.

### Medium impact SoVs

Any location of sensor placement, for example at the wrist, foot, ankle, lower back, and chest, offers a distinct trade-off between comfort and variability. For example, if the measurement process requires the estimation of a walking parameter such as speed and stride length, a cumbersome foot-mounted sensor will produce the highest accuracy measurement with the least amount of variability; a lower back or chest-mounted strap would result in high to moderate accuracy and variability; and, a widely available wrist sensor would produce the least accurate and most variable information^22,23^. Overall, variability increases as the distance between sensor and body location of interest increases, which implies that mitigation strategies should aim to minimise separation between the two locations. For example, using a foot rather than wrist sensor when a subject walks while holding a phone, will clearly offer significantly greater accuracy in gait parameter estimation. Nevertheless, practical sensor placement may be influenced by accessibility, subject comfort, and even cultural norms. When the preferred location is not available, careful algorithm selection can help reduce the influence of this SoV^24^.

Arguably, next to location the second most influential SoV is the orientation of the sensor on the body. Accelerometers in particular are extremely sensitive to changes in orientation: For example, a typical accelerometer with a range of + /− 8 g and a 10-bit analog-to-digital converter (ADC) will have a resolution of approximately 1.4 degrees. A 10-degree body position change will produce a bias of 0.06 g change in acceleration. Large orientation variations can be expected in practice, sensors are not always precisely placed by the subject, or sensors can erroneously be placed upside down, backward, or at an odd angle, resulting in a large constant bias. While a constant bias can be estimated and removed by a high-pass filter set at a very low frequency, for example, 0.25 Hz, non-constant bias is much more challenging to remove. Non-constant bias due to frequent orientation changes is especially likely to occur when a sensor is attached over clothing on a body part with high mobility such as the wrist, or a large muscle group such as the quadriceps femoris, or when a sensor is loosely affixed to the body. This can result in significant localized movement and rotation of the sensor relative to the body during data collection resulting in significant fluctuations in the signal which can significantly lower the signal fidelity. Remediation of this SoV is to ensure that the measurement process provides specific guidance such as all sensors be fixed tightly on the body underneath articles of clothing to minimize relative movement, especially when placed on a hyper-mobile body part, such as the wrist.

Further mitigation of orientation SoVs is possible through the use of orientation-invariant algorithms^25^. A common approach is to first estimate the true sensor orientation on the body, and subsequently calculate the rotational offset between the actual and the “ideal” sensor orientation using standard mathematical transformations^26^ and subsequently to employ orientation-invariant correction algorithms. Alternatively, selecting orientation-agnostic features where possible, such as those derived in the frequency domain, would effectively eliminate variability from orientation. Further, it is conceivable to investigate the influence of sensor placement and orientation on sensor data, sensor data features, and digital biomarkers using a novel biomechanical simulations method introduced by Derungs and Amft^27^.

### High-impact SoVs

While low- and medium-impact SoVs have established mitigation strategies, high-impact SoVs require careful consideration and may require auxiliary exploratory studies to investigate and quantify their influence and hence may require considerable additional effort for the development of appropriate mitigation measures.

### Data-driven investigation of SoV impact: case studies

Although it is often possible to use published literature to assess the impact of SoVs, certain settings require the clinical research team to explore specific SoVs within a particular Context of Use and with reference to specific outcome measures. In this Section, we present two case studies that follow a data-driven approach to investigate the potential impact of particular choices in the measurement process. The work presented below is not intended as a comprehensive investigation of the specific SoVs considered, but rather, as a way to illustrate a practical approach to assess their impact at the pilot stage of clinical research. Our analysis is focused on practical ways to identify relevant SoVs of concern before committing to a clinical study protocol design. When specific SoVs are identified as potentially high-impact a full follow-up investigation would be required for example by modelling their impact in terms of erroneous diagnosis.

### Case study 1: Device type, number of sensors, and sampling rate

**Fig. 1:**
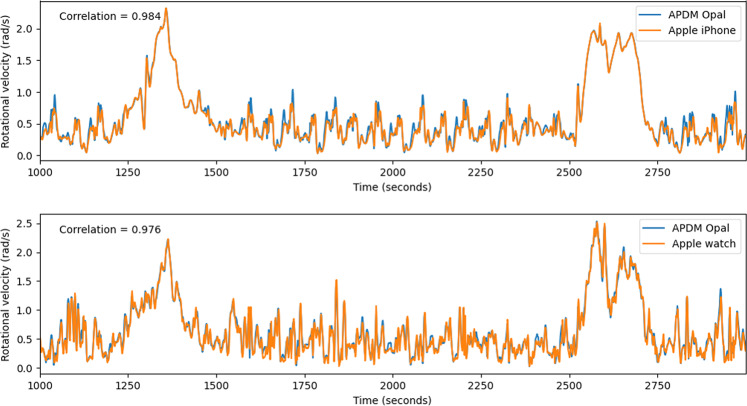
Angular Velocity Comparison. Top: Comparison of angular velocity calculations using APDM Opal (blue) and iPhone (orange) samples with both devices placed at lumbar region. Bottom: Comparison of angular velocity calculated using Opal (blue) and Apple Watch (orange) samples with both devices placed on the same wrist of the subject.

Using data collected during exploratory in-clinic piloting of WATCH-PD (cf. Methods section below and Adams et al.^28^, we investigate variability across device types including a popular consumer platform, and differences due to their placement, sampling rate and sampling locations. To compare consumer- (Apple Watch and iPhone) against research-grade (APMD Opal) devices, we analysed data recorded during a one-minute-walk task, where both devices were simultaneously employed (APMD Opal sensors were placed under the Apple device). Figure 1 shows angular velocity calculations obtained from gyroscope data from several subjects, comparing Opal against Apple Watch and iPhone. Opal data were recorded at 128 Hz, down-sampled and time-shifted to align with Apple Watch measurements (bottom) and separately with iPhone (top). Figure 1 suggests that both consumer-grade devices reproduce high, low, and intermediate frequencies at comparable quality to the Opal reference (with correlation of 0.984 and 0.976 correspondingly).

**Fig. 2:**
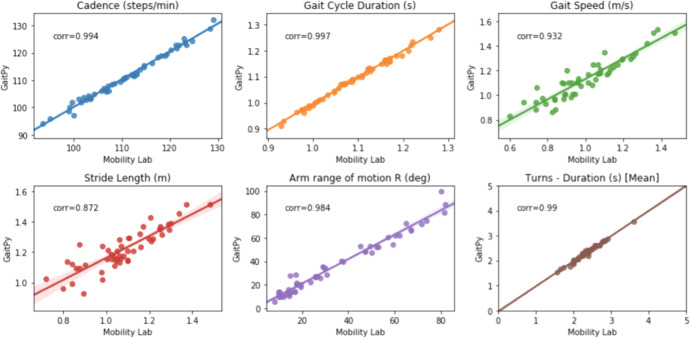
Gait Features Comparison. Correlation between gait features calculated on the same measurements performed using Opal and using GaitPy and Mobility Lab correspondingly.

**Fig. 3:**
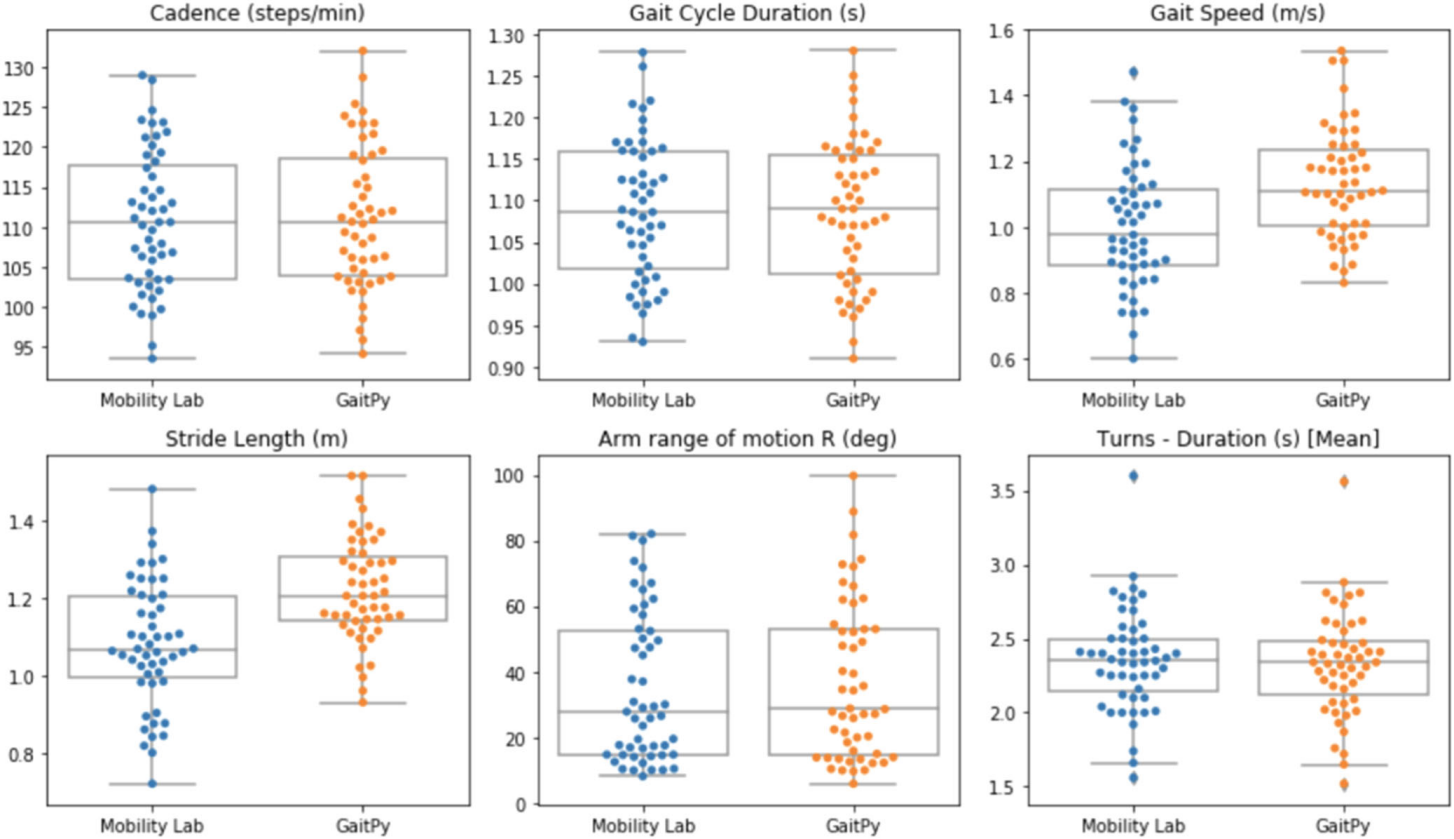
Gait Feature Variability. Variability of selected gait features calculated on the same measurements performed using Opal and using GaitPy and Mobility Lab correspondingly. The solid line represents the median value; the box limits show the interquartile range (IQR) from the first (Q1) to third (Q3) quartiles; the whiskers extend to the furthest data point within Q1–1.5*IQR (bottom) and Q3 + 1.5*IQR (top).

Further, we compared gait features obtained from Opal measurements using the Mobility Lab software provided by APDM (cf. https://apdm.com/mobility/) now part of Clario, against iPhone data processed using software developed in-house (by co-author TRH). The latter, employs the El-Gohary et al.^29^ algorithm to identify gait bouts after turns, and subsequently extract gait features using GaitPy^30^ following the approach suggested by ref. ^31^. In-house developed software (also by TRH) was used to compute rotational velocity at the wrist during arm swings per gait cycle. Figure 2 suggests very strong agreement between the two approaches across all features (with correlation of cadence, gait arm range and turns exceeding 0.9). Figure 3 further suggests that both approaches result into comparable levels of variation in all features. However, gait speed and stride length appear to produce significant (but consistent) differences in absolute terms. This is caused by the use of per-subject height and leg-length measurements obtained at enrolment in Mobility Lab calculations, while in GaitPy a fixed height-to-leg-length factor is employed across all subjects. The latter clearly limits the accuracy of the pendular model employed by in the calculation of these features.

**Fig. 4:**
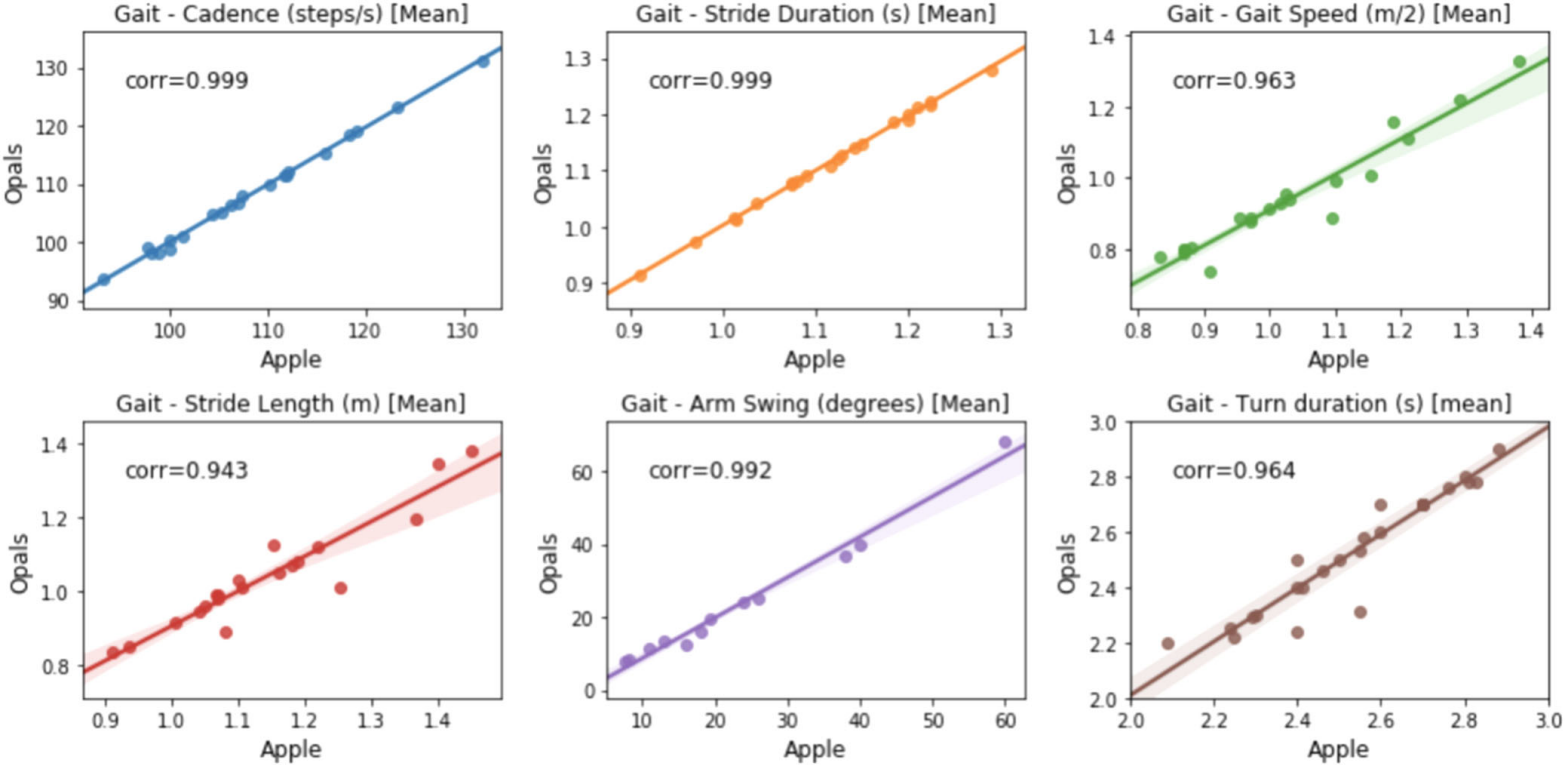
Comparison against Consumer-grade Devices. Comparing features estimated using Opal versus consumer-grade devices using the software developed in-house. All features use iPhone measurements except arm swing that employs data sampled using the Apple Watch.

Finally, in Fig. 4 gait features estimated using Opal measurements are compared against measurements from consumer- devices using the software developed in-house (signals were aligned as described above). While there is still strong agreement overall, there are also noticeable differences. One cause for this mismatch is likely to be due to the small angular misalignment introduced by the specific placement of the devices on top of each other as described above.

**Fig. 5:**
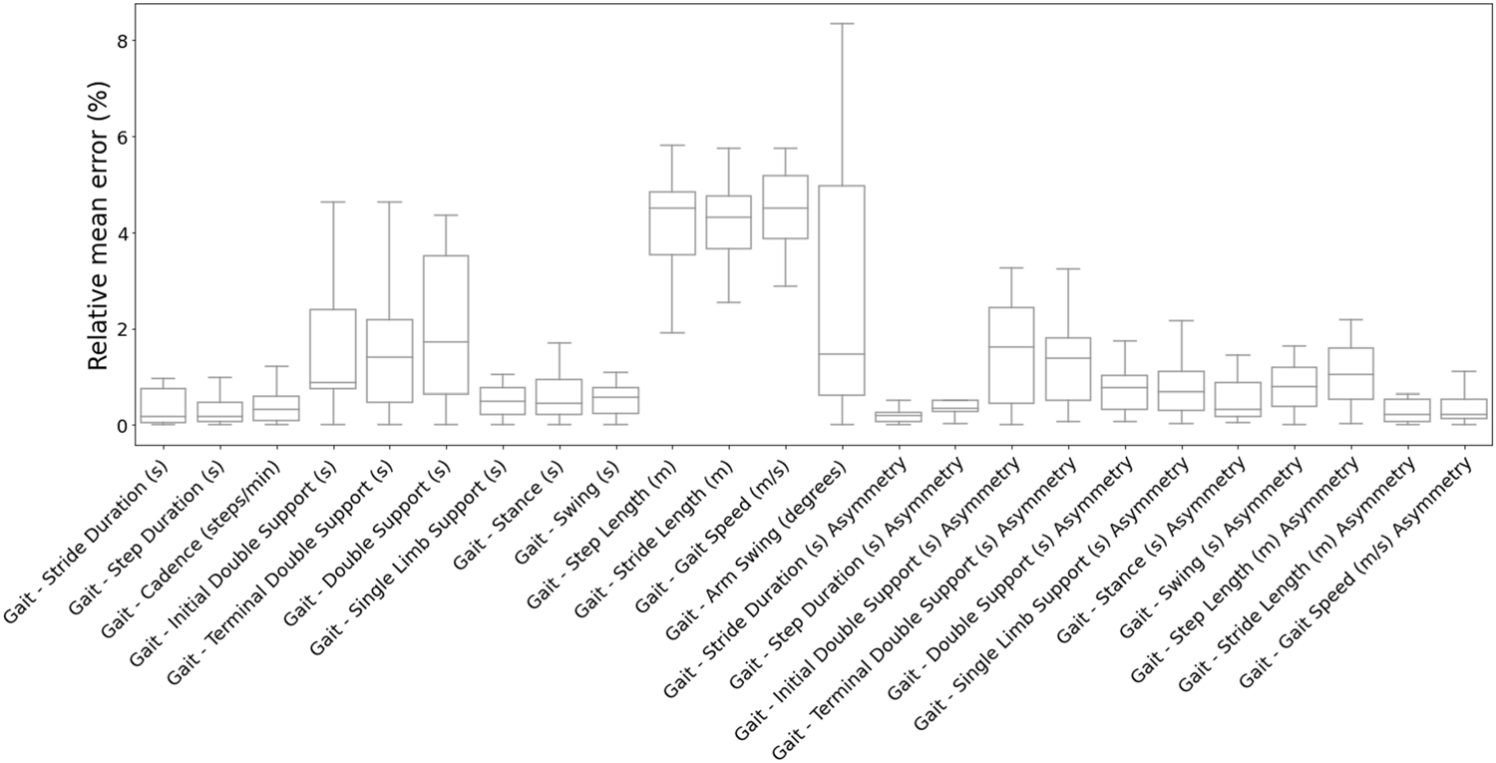
Variability of Gait Features. Distribution of the relative mean error across 50, 100 and 128 Hz sampling rates per calculated feature. The solid line represents the median value; the box limits show the interquartile range (IQR) from the first (Q1) to third (Q3) quartiles; the whiskers extend to the furthest data point within Q1–1.5*IQR (bottom) and Q3 + 1.5*IQR (top).

To explore sampling frequency as a SoV, Opal measurements were down-sampled to obtain data at 50 and 100 Hz. Figure 5 demonstrates the limited impact of lower sampling rates on feature estimation in terms of error. Features involving double support and asymmetry are most affected because they are more sensitive to error propagation caused by small inaccuracies in the calculation of underlying metrics. This interpretation is supported by the findings of ref. ^32^, which conducted an extensive evaluation of seven different IMUs: Accelerometer and gyroscope data from each device were processed using the same algorithm and compared against ground truth obtained using OptoGait (cf. http://optogait.com). Similar to our analysis, temporal parameters demonstrated less variability to spatial parameters for which more complex calculations are needed for, example, double integration and an error-state Kalman filter, and are thus sensitive to even small measurement inaccuracies. Zhou et al.^32^ traced the latter to device issues such as insufficient ADC range or inadequate sensor calibration. Overall, our investigation suggests that features less sensitive to low-frequency sampling can be identified using the above observations as appropriate for the specific Concept of Interest and Context of Use.

### Case study 2: Environmental Factors

**Fig. 6:**
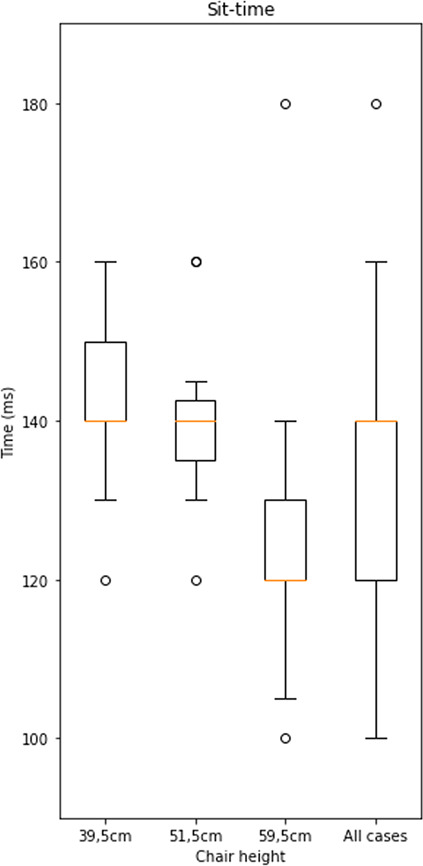
Variability due to Chair Selection. Chair height as a SoV for sit-to-stand transition time tests. Variability is considerably larger when considered across chairs. The error bars of boxplot are generated by matplotlib using the matplotlib.pyplot.boxplot function with default parameters. The boxplot extends from the first to the third quartile of the data with a line at the median. The whiskers extend from the box by 1.5 times the inter-quartile range.

Variability due to environmental factors, that is, factors relating to the setting within which the measurement process is performed, rather than the process per se, can also affect outcome measures. For example, Perraudin et al.^33^ identified the height of the chair used to perform sit-to-stand transition time tests as a key environmental SoV in this context. Using data provided by the DOMVar project obtained from an actigraphy bracelet incorporating gyroscope and accelerometer (cf. Methods Section below), Fig. 6 illustrates the effect of three chairs of different heights (39.5 cm, 51.5 cm, and 59.5 cm) on average across 12 transitions for each of three subjects. Note the significantly higher variability when data are aggregated across chair heights. Hence, passive monitoring at a patient’s home, where typically different chair types would be present, is likely to result into less consistent outcome measure estimation.

**Fig. 7:**
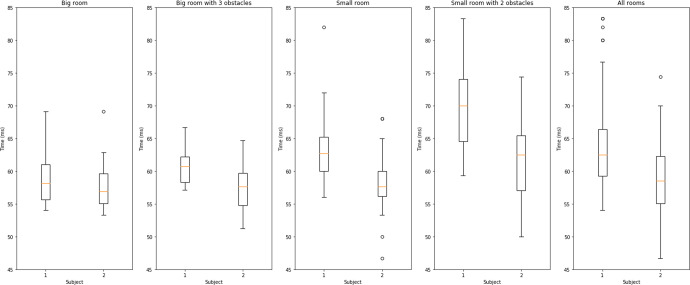
Stride Time Variability. Stride time variability arising from the home environment. The panels from left to right show box plots of passively recorded stride time for two subjects in four different settings and in aggregate. The error bars of boxplot are generated by matplotlib using the matplotlib.pyplot.boxplot function with default parameters. The boxplot extends from the first to the third quartile of the data with a line at the median. The whiskers extend from the box by 1.5 times the inter-quartile range.

Further, walking speed can be strongly influenced by room size and the arrangement of furniture within in. Figure 7 illustrates stride time variability for two healthy subjects. Data is collected passively using the actigraphy bracelet in four different settings, namely: (i) large empty room, (ii) large room containing furniture obstacles, (iii) small empty room, and (iv) small room containing furniture obstacles.

Both examples above suggest that passive monitoring, in particular, is especially sensitive to environmental factors. When the passive monitoring is preferable for clinical reasons, averaging the relatively larger number of measurements can reduce variability when no systematic changes in the SoVs are expected, for example, when the layout of the patient’s home changes to accommodate further restrictions in their movements their symptoms progress.

## Discussion

In this paper, we introduced a conceptual framework for the identification and characterisation of SoVs related to the use of DHTs in clinical trials for PD. We distinguish SoVs related to experimental design and choice of technology against variability introduced by the subject, either inherently or due to the disease. This framework aims to provide practical guidance on how to investigate, assess, and where possible, mitigate their influence on the measurement process targeting a particular Concept of Interest in a specific Context of Use.

To this end, the choices between active or passive monitoring and the duration of the study are especially influential. In our experience, investigators often incorporate elements of both active and passive assessments despite the lack of due justification. Active approaches are often sufficient to provide conclusive evidence and achieve higher specificity of the derived outcomes measures. For example, an active approach to quantify movement quality would be less likely to be affected by environmental factors (Case Study #2). However, passive monitoring would be preferable when the relevant Concept of Interest is associated with the subject’s overall patterns of movement, such as general long-term activity levels or the quantification of relatively rare events such as falls and freezing. Indeed, in the case of falls and freezing, active assessment would likely be ineffective despite its lower variability, due to the lack of sufficient motor performance variability during measurement periods^34^. A pragmatic approach is to view active assessment as more suitable to the measurement of subject capacity and passive assessment as a mechanism to capture real-life ability^35^. The above observation does not preclude adopting a hybrid approach if necessary. In this case, the presented framework and case studies still offers a useful guide to determine the potential influence of SoVs on study-specific Concepts of Interest.

Further, a key motivation in initiating this work was the need to clearly contrast variability due to the measurement process against variability caused by the disease. To this end, we believe that a core requirement towards the further development of mitigation techniques for a wider range of SoVs is to place greater emphasis on normative data sets reflecting performance by healthy subjects. This information is critical to establish ground truths of expected variability.

Finally, an inherent characteristic of DHTs is the rapid rate of advance in sensor technologies and the ability of modern software tools, such as machine learning and artificial intelligence, to improve their quantitative performance. Such rapid innovation can exacerbate the impact of SoVs, for example hardware used in a prospective clinical study might become outdated by the time the study is finished; or, algorithm performance might be enhanced by updating the software mid-study based on additional training data. Clearly, SoVs introduced by the availability of improved tools must also be managed in adopting a similar approach to the suggested SOV framework presented here. Alternatively, requiring new prospective studies for every major hardware, firmware, or model upgrade would represent a major barrier to innovation.

## Methods

### 3DT working group on SoVs

Created in partnership with Parkinson’s UK, the Critical Path for Parkinson’s Consortium (CPP) is a global initiative supporting collaboration among scientists from the biopharmaceutical industry, academia, government agencies, and patient-advocacy associations. The value of such collaborations is recognized by global regulatory agencies, including the US Food and Drug Administration and the European Medicines Agency, which have actively encouraged data-driven engagement through multi-stakeholder consortia^36^. A foundational tenet of CPP is the precompetitive collaborative nature of the consortium that forms the core principle for advancing the regulatory maturity of DHTs, and thus, facilitate their use in future clinical trials. To this end, CPP established the Digital Drug Development Tool (3DT) project, a precompetitive collaboration, aiming to align knowledge, expertise, and data sharing of DHTs across its consortium. Its main goal is to complement standard clinical assessments with a set of candidate objective digital measures, which can provide high precision measurements of disease progression and response to treatment.

This paper reports on the findings of the CPP 3DT: Sources of Variability (SoVs) Working Group. To develop the conceptual framework for the identification and characterisation of SoVs presented here, the WG adopted a triangulation methodology incorporating findings reported in the current research literature, direct experience with proprietary or unpublished work contributed by individual WG members, and data-driven analysis of key cases studies identified.

### Data sets

Data used in Case Study 1 were pilot data obtained from Wearable Assessments in the Clinic and Home in PD (WATCH-PD), a 12-month multicentre, longitudinal, digital assessment study of PD progression in subjects with early untreated PD (clinicaltrials.gov#: NCT03681015). Its primary goal is to generate and optimize a set of candidate objective digital measures to complement standard clinical assessments in measuring the progression of disease and the response to treatment. A secondary goal is to understand the relationship between standard clinical assessments, research- grade digital tools used in a clinical setting, and more user-friendly consumer digital platforms to develop a scalable approach for objective, sensitive, and frequent collection of motor and nonmotor data in early PD. The clinical protocol^28^ includes: (a) in-clinic assessments using six APDM Opal inertial measurement unit (IMU) sensors^37^ that are placed in the lumbar region, sternum, wrists, and feet of the subject, which record accelerometer and gyroscope signals during a series of mobility-related tasks; and (b) a walking task performed at-home, where patients are instructed to place an iPhone in a pouch provided, and attach it to the lower back, and then initiate sensor data recording using an Apple Watch. The WATCH-PD trial has been approved by the WIRB Copernicus Group (protocol code WPD-01 and date of approval 12/21/2018). Informed consent was obtained from all subjects involved in the study. Written consent will not be obtained from participating participants since they are not identifiable by the study team. Participants are only identifiable at the study site level.

The data set used in Case Study 2 was obtained during software testing (quality improvement and usability) by the Digital Outcome Measure Variability due to Environmental Context Differences using Wearables project (DOMVar), conducted collaboratively between Birkbeck College, University of London, University College London and Panoramic Digital Health (who provided the study device cf. https://www.panoramicdigitalhealth.com/). The project was conducted according to The European Code of Conduct for Research Integrity (2017) and the guidelines of the Code of Practice on Research Integrity of Birkbeck College, University of London, and approved by the Ethics Committee of Birkbeck College, University of London. Informed consent was obtained from all subjects involved in the study.

### Reporting summary

Further information on research design is available in the Nature Research Reporting Summary linked to this article.

## Supplementary information

Reporting Summary

## Data Availability

Software quality testing data used in Case Study 2 are available to qualified researchers from co-author DH. Data presented in in Case Study 1 is from the ongoing WATCH-PD study and cannot be shared until completion and dissemination of results and approval by study sponsor. This is expected to become possible within 24 months from the acceptance date of this paper. Qualified researchers will be able to contact co-author RD at the University of Rochester, to request access to the data.

## Code availability

Python source code of the custom software used to conduct the calculations presented in Case Studies 1 and 2 is available upon reasonable request from co-authors KPK and DH correspondingly. In Case Studies 1, the APDM Mobility Lab software (cf. https://apdm.com/mobility/) now part of Clario, was used with default parameters. Co-author TRH developed the custom software employed using python and incorporating the GaitPy module. GaitPy was implemented following the parameters suggested by ref. ^31^.
